# Case Report: Triphasic Waves in a 9-Year-Old Girl With Anti-NMDAR Encephalitis

**DOI:** 10.3389/fneur.2022.819209

**Published:** 2022-01-25

**Authors:** Ke Zhang, Shuang Xu, Yalan Zhou, Tangfeng Su

**Affiliations:** Department of Pediatrics, Tongji Hospital, Tongji Medical College, Huazhong University of Science and Technology, Wuhan, China

**Keywords:** triphasic waves, EEG, anti-NMDAR encephalitis, pediatric, metabolic encephalopathy, case report

## Abstract

**Background:**

Triphasic waves (TWs) are mainly described in association with metabolic encephalopathy, especially hepatic encephalopathy. Now, as different conditions including non-metabolic and structural abnormalities have been reported to be associated with TWs, the presence of TWs becomes a non-specific finding for metabolic encephalopathy.

**Case Presentation:**

We report the first case of anti-NMDAR encephalitis in a 9-year-old girl presenting with TWs on EEG. The TWs background EEG lasted for about 12 h on the 40th day of the disease course. No epileptic wave was found during a series of EEG examinations. The child was discharged from the hospital and no neurological sequelae remained after a six-month follow-up.

**Conclusions:**

TWs are not specific to metabolic encephalopathy, but can also occur in children with autoimmune encephalitis. This case achieved a good prognosis after the early initiation of immunotherapy.

## Introduction

Background EEG patterns (non-convulsive status epilepticus (NCSE), non-convulsive seizures (NCS), periodic discharges, and triphasic waves) play an important role in the diagnosis and management of patients presenting with encephalopathy in the intensive care unit. Encephalopathy may be secondary to infectious, metabolic and autoimmune etiologies. EEG should be considered as a good tool to assess the functional status of the brain in patients with encephalopathy, especially for prognostic purposes.

Triphasic waves (TWs) are abnormal EEG waveforms, first described as “blunted spike and wave” in patients with hepatic encephalopathy by Foley in 1950 (1). Later, Bickford and Butt proposed the term “triphasic waves” according to the characteristic shape of the waves in 1955 (2). Now, TWs refer to generalized periodic discharges (GPDs) with triphasic morphology for brevity in the American Clinical Neurophysiology Society (ACNS) classification and the revised glossary and updated proposal for EEG terms (3). The typical TWs are composed of three phases: a high-amplitude positive wave preceded and followed by a slow negative deflection, usually occurring periodically in the frequency range of 1.5 to 2 HZ, predominantly in the frontal and central regions. A fronto-occipital lag ranged from 25 to 140 milliseconds can be observed (4). A wide variety of pathological conditions have been reported to be associated with TWs, including hepatic, renal, hypoxia, structural, toxic and metabolic abnormalities (5).

It is worth noting that TWs are not common in people under the age of 30, especially rarely recorded in children (6). Here, we report the first case of anti-NMDAR encephalitis in a young child presenting with TWs on EEG.

## Case Presentation

A 9-year-old girl with uncontrollable twisting of lower limbs was admitted to the pediatric intensive care unit due to a depressed level of consciousness. One month ago, the child developed frequent twitching of the left lower extremities for 1–2 h, with no change in her level of consciousness during those attacks. Except for the abnormal limb movements, her parents complained the child recently had mental disorders, manifesting as low frustration threshold, worse memory, and bouts of involuntary laughter. She was then diagnosed with mental illness by a local hospital after the exclusion of conceivable organic medical conditions and was treated with aripiprazole, lorazepam, magnesium valproate and sertraline hydrochloride. However, involuntary muscle twitches were not alleviated but became more frequent and extended to other body regions.

**Figure 1:**
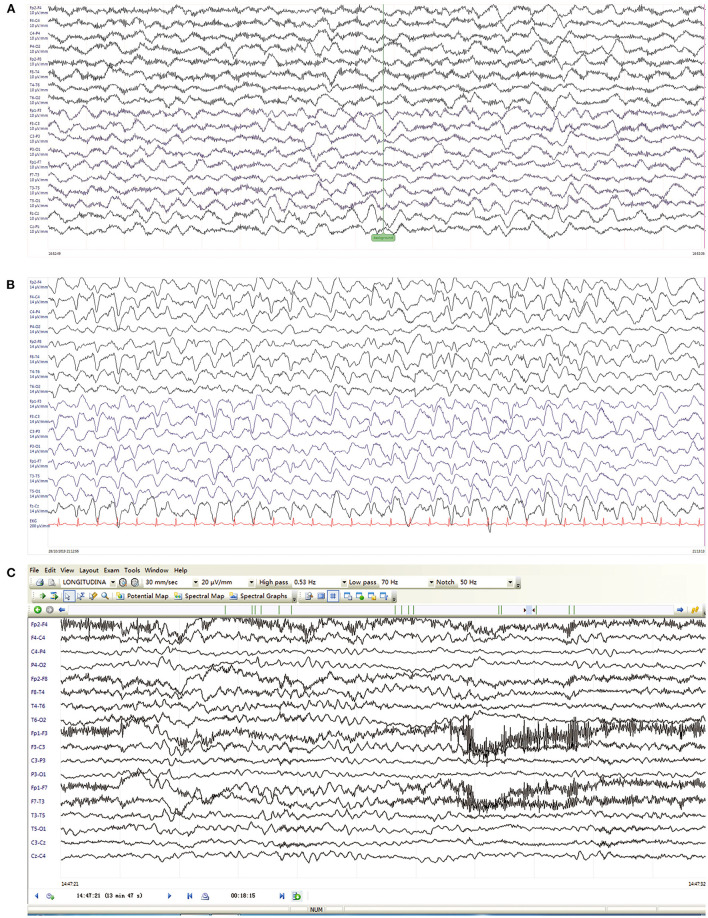
EEG on day 2 **(A)**, day 8 **(B)** after PICU admission, and six months after inpatient discharge **(C)**. **(A)** Day 2, EEG revealed diffuse irregular slow waves. **(B)** Day 8, EEG showed generalized triphasic waves predominantly in frontal regions. **(C)** Six months later, EEG demonstrated mild slow background.

At admission, the patient exhibited a stuporous mental state, with a Glasgow Coma Scale score of 11 (E3M3V5). Neurological examination revealed no nuchal rigidity, Brudzinski's sign or Kernig's sign. Laboratory tests, including complete blood cell counts, liver and renal functions, serum electrolyte, ammonia, and blood glucose, were all within normal limits. Cranial MRI also revealed no specific abnormality. A subsequent lumbar puncture demonstrated a pressure of 221 mmH2O (80-180 mmH2O), while the routine and biochemical CSF tests showed normal results. Results of the study for microbiological investigations on CSF were negative, including CSF microscopy, CSF culture, CSF serology (herpes simplex virus [HSV] IgG/IgM, cytomegalovirus [CMV] IgG/IgM, varicella-zoster virus [VZV] IgM, measles IgM, Epstein-Barr's virus [EBV] IgM) and polymerase chain reaction (PCR) analysis for neurotropic viruses. EEG on day 2 showed diffuse slow waves without epileptiform discharges (Figure 1A). These results suggested a low probability of infectious causes. Besides, the child underwent examination of ultrasound and CT scans, which showed no teratoma or other tumors.

**Figure 2:**
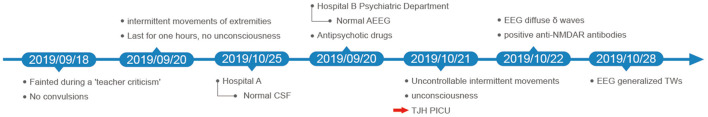
Timeline of the clinical course.

Based on the typical clinical features (rapid onset, psychiatric symptoms, movement disorder, decreased consciousness and abnormal EEG), autoimmune encephalitis was highly suspected, which was later confirmed by positive anti-NMDAR antibodies in serum and CSF. Empirical therapy was immediately administered with intravenous methylprednisolone (500 mg/d) and gamma globulin (12.5 g/d). A 24-h video EEG on day 8 revealed generalized TWs more prominent anteriorly lasting for about 12 h (Figure 1B and timeline in Figure 2). In addition, the child's frequent involuntary jerking movements were confirmed as non-epileptic seizures by the long-term video EEG monitor. No signs of metabolic dysfunction were displayed. Although long-term video EEG recordings showed a disappearance of TWs the next day, the child's mental status still did not improve. Assessment of the child's consciousness revealed partial recovery on day 17, while simultaneous EEG showed diffuse slow waves. The patient finally recovered and was discharged from hospital on day 37 without any evidence of neurological sequelae. Six months later, a routine EEG follow-up just showed mild slow background activity (Figure 1C).

## Discussion

Autoimmune encephalitis (AE) is an inflammatory disease of the central nervous system mediated by autoimmune disorders. The main clinical manifestations usually include abnormal mental behavior or cognitive impairments, decreased levels of consciousness, speech dysfunction, dyskinesia, seizures, autonomic nervous dysfunction and central hypoventilation. Anti-NMDAR encephalitis is the most common type of AE, accounting for 4% of all encephalitis (7).

EEG plays an important role in patients with AE, not only for detecting seizures, assessing prognosis, but also for diagnosis in some cases. Almost all patients with AE are found with abnormal EEG findings, which usually showing non-specific diffuse slowing of the background, focal slowing and sometimes accompanied by variable epileptiform activity. Moise et al. described the continuous EEG findings of 64 patients with AE, with generalized rhythmic delta activity more commonly seen in anti-NMDAR encephalitis. They also identified extreme delta brush (EDB) as a signature EEG pattern in anti-NMDAR encephalitis (8). Zhang et al. conducted a study that addressed the EEG features of 34 children with anti-NMDAR encephalitis. EEG characteristics before immunotherapy included generalized slowing (73.5%), focal slowing (5.9%), normal background activity (20.6%) and interictal epileptic paroxysms (47.1%). EDB were recorded in 2 patients (9). EDB, characterized by beta bursts overriding on delta waves in EEG analysis, has been currently considered to be pathognomonic in 5–33% of patients with anti-NMDAR encephalitis, most in more severe cases and with poor prognosis (10). Although a variety of EEG abnormalities associated with anti-NMDAR encephalitis have been reported, to the best of our knowledge, this is the first case report of anti-NMDAR encephalitis presenting with TWs on EEG in a child.

TWs are usually described in association with metabolic encephalopathy, especially hepatic encephalopathy. However, a growing body of case reports indicate that TWs may also occur secondary to other etiologies, such as uremia, hypernatremia, hyponatremia, hypercalcemia, hypoglycemia, hyperthyroidism, hypothyroidism, drug-induced and anoxic encephalopathy. Other causes linked with TWs include stroke, tumors, Creutzfeldt-Jakob disease and dementia (6, 11). Among inflammatory and infectious encephalopathies, TWs are reported in encephalopathy of sepsis, Mollaret's meningitis, herpes simplex encephalitis, Borrelia burgdorferi meningoencephalitis, tuberculous meningitis, carcinomatous meningitis and Hashimoto's encephalopathy (12–14). To our knowledge, only one study reported a case of EEG with TWs in an AE adult case (15).

Although common in adults, TWs are relatively rare in children. Janati et al. reported two cases of TWs in drowning children (16). Hosain et al. analyzed EEGs in 178 children with coma and found that TWs appeared in only 6 children (17). Laan et al. described TWs over the frontal regions on EEG in children with Angelman syndrome, a rare genetic disorder (18). In our case, a 9-year-old girl presented with TWs during the course of anti NMDAR encephalitis.

In view of the numerous causes of TWs, metabolic encephalopathies should be considered first. In our case, the blood ammonia, glucose and electrolyte were at normal levels, so that metabolic encephalopathy could be basically excluded. In addition, TWs have also been described in toxic encephalopathy induced by valproic acid, cefepime, cefoperazone, ceftriaxone, lithium, and pregabalin (19–21). Vulliemoz et al. also reported TWs in a patient with chronic renal failure after administration of levetiracetam (22). Our patient had initially taken valproic acid on admission, but soon changed to levetiracetam. In addition to normal hepatorenal function and no history of the above medicines, the possibility of toxic factors was small. Furthermore, seizure is a common clinical manifestation in children with anti NMDAR encephalitis, and in some cases, NCSE could be present. Dilaver et al. described 15 cases aged 66 ± 8 years with NCSE whose EEGs presented with atypical triphasic waves (23). Sometimes, it is difficult to attribute the presence of TWs to NCSE or encephalopathy. Benzodiazepines (BZPs) or non-sedating anti-seizure drugs (NSAEDs) are commonly administered to differentiate them (24). However, there is debate over the utility of the trials, as the administration of BZPs or NSAEDs may also diminish TWs in metabolic/toxic encephalopathies (25). Nevertheless, patients' mental status should be specially paid attention to after the therapy of BZPs or NSAEDs. In NCSE, both epileptiform discharges and consciousness can be improved or normalized with the medicines, whereas the mental state is not improved in metabolic/toxic encephalopathies (26). Owing to dyskinesia, midazolam was administered to the girl with continuous infusion upon admission, even during the EEG monitor, which signified this EEG pattern occurred in spite of the use of BZPs. Of note, TWs background in this patient was monotonous and did not show highly changing and time-dynamic spatiotemporal evolution. Besides, after TWs disappeared, the child remained comatose. We tend to believe that the TWs on the EEG were indeed due to anti-NMDAR encephalitis.

The mechanism of TWs is still unclear. Bickford et al. considered TWs as a positive traveling wave along the cortex, caused by subcortical disturbance at the thalamocortical level (2). Gloor P et al. suggested that the abnormal oscillatory discharges between cortical and thalamic neurons may be responsible for this EEG pattern (27). Now, it is supposed that TWs may result from metabolic or structural abnormalities at the thalamocortical level, especially in the thalamocortical relay neurons. Abnormal glutamate metabolism is probably one of the mechanisms.

There are different arguments about the prognosis of TWs. Bahamon-Dussan J. E. et al. studied 30 patients with TWs, all of whom had different degrees of altered mental status. After 22 months of follow-up, the overall mortality was 77%. Only three of the seven survivors had normal nervous systems. They concluded that TWs indicated a dismal prognosis (28). Sutter et al. analyzed the EEGs of 154 patients with encephalopathy and found TWs implied higher mortality (29). Whereas, Kaplan et al. believed that the prognosis often depended on the underlying causes. If TWs occurred in the setting of reversible toxic metabolic dysfunction, the outcome could be better. If in the cases of closed head injury or hypoxia, the prognosis could be poor (30). Sutter et al. later followed up 105 patients with TWs. They suggested that the unresponsiveness of EEG, rather than the underlying etiology, could be an independent factor associated with high case fatality rate (31). In our case, the primary disease of the girl was anti-NMDAR encephalitis, having a recovery rate of ~80% (32). Besides, her EEG showed good reactivity. Both the primary disease and EEG reactivity predicted a favorable outcome.

## Conclusion

Our case report further confirms that TWs are not specific to metabolic encephalopathy, but can also occur in children with autoimmune encephalitis. More importantly, the possibility of metabolic or drug-induced encephalopathy should be first ruled out when TWs appear. To our knowledge, this is the first report of TWs observed in a child with anti-NMDAR encephalitis.

## Data Availability Statement

The original contributions presented in the study are included in the article/supplementary material, further inquiries can be directed to the corresponding author/s.

## Ethics Statement

The studies involving human participants were reviewed and approved by the Ethics Committee of Tongji Hospital, Tongji Medical College, Huazhong University of Science and Technology. Written informed consent to participate in this study was provided by the participants' legal guardian/next of kin. Written informed consent was obtained from the individual(s), and minor(s)' legal guardian/next of kin, for the publication of any potentially identifiable images or data included in this article.

## Author Contributions

KZ prepared the original draft. SX and YZ revised the manuscript. TS reviewed and edited the final manuscript. All authors contributed to drafting or revising the article, gave final approval of the version to be published, and agree to be accountable for this work.

## Conflict of Interest

The authors declare that the research was conducted in the absence of any commercial or financial relationships that could be construed as a potential conflict of interest.

## Publisher's Note

All claims expressed in this article are solely those of the authors and do not necessarily represent those of their affiliated organizations, or those of the publisher, the editors and the reviewers. Any product that may be evaluated in this article, or claim that may be made by its manufacturer, is not guaranteed or endorsed by the publisher.
